# Kernel partial diagnostic robust potential to handle high-dimensional and irregular data space on near infrared spectral data

**DOI:** 10.1016/j.heliyon.2020.e03176

**Published:** 2020-01-31

**Authors:** Divo Dharma Silalahi, Habshah Midi, Jayanthi Arasan, Mohd Shafie Mustafa, Jean-Pierre Caliman

**Affiliations:** a SMART Research Institute, PT. SMART TBK, Riau, Indonesia; b Institute of Mathematical Research, Universiti Putra Malaysia, Serdang, Malaysia

**Keywords:** Analytical chemistry, Near infrared, Spectral data, Partial least squares, Diagnostic robust generalized potential, Nonlinear, Kernel, Hilbert-space

## Abstract

In practice, the collected spectra are very often composes of complex overtone and many overlapping peaks which may lead to misinterpretation because of its significant nonlinear characteristics. Using linear solution might not be appropriate. In addition, with a high-dimension of dataset due to large number of observations and data points the classical multiple regressions will neglect to fit. These complexities commonly will impact to multicollinearity problem, furthermore the risk of contamination of multiple outliers and high leverage points also increases. To address these problems, a new method called Kernel Partial Diagnostic Robust Potential (KPDRGP) is introduced. The method allows the nonlinear solution which maps nonlinearly the original input X matrix into higher dimensional feature mapping with corresponds to the Reproducing Kernel Hilbert Spaces (RKHS). In dimensional reduction, the method replaces the dot products calculation of elements in the mapped data to a nonlinear function in the original input space. To prevent the contamination of the multiple outlier and high leverage points the robust procedure using Diagnostic Robust Generalized Potentials (DRGP) algorithm was used. The results verified that using the simulation and real data, the proposed KPDRGP method was superior to the methods in the class of non-kernel and some other robust methods with kernel solution.

## 1 Introduction

The Near Infrared Spectroscopy (NIRS) technology as secondary measurement has been widely used in varies agricultural process. It requires less (even no) sample preparation, non destructive, rapid, and chemical-free with its great potential for product quality control and real time analytical chemistry process. The NIRS uses a spectrometer to collect a continuous absorption spectrum. This spectrum associates with the chemical and physical composition of the sample material. Practically, NIRS applies the multivariate statistics method on the spectral dataset to build the fitted model. The model is built by associating the spectral dataset measured from the known sample with a set of reference data. In routine chemical analysis, this model is applied in the analytical process to predict the concentration of the unknown samples.

The NIR spectral data is presented as a high dimensional matrix. It contains wide wavelengths ranges (350–2500nm: 1 or 2nm interval) as predictor variables and takes a huge number of samples in line with the increasing volumes of routine laboratory analysis. Normally, each spectrum NIR has more than thousands data points. Based on experience, the collected spectra also may often composed of complex overtone and many overlapping peaks that result in broad NIR absorption bands [1]. These are caused by the physical properties, chemical variation, and the instrumentation which lead to misinterpretation on the spectra information. This makes the spectral data processing becomes more complicated. To encounter this, a well assign of multivariate calibration based on statistical methods are highly considered.

In multivariate calibration, the Partial Least Squares Regression (PLS) method [2] is the most used. The PLS has benefit to tackle the presence of multicollinearity and high dimensional in the dataset. In fact, in the application the method is still less inferior due to its efficiency and interpretability. The complexity in the NIR spectral dataset is also an issue, where it may cause the risk of involving multiple outliers and high leverage points increases. This may decrease the effectiveness in the computational. As solution, then the robust procedure is needed to apply with the PLS method.

The PLS works by projecting the X matrix to a smaller set of uncorrelated variables called new components with corresponds to y vector. It is known as a class of linear methods with the basic linearity assumptions holds both in the relation between observations and for modeling in the latent variables [3]. However, in the real NIR spectral dataset the sets of predictor matrix X and response matrix y could have a nonlinear relationship. This is due to the scattering effect and noise problems in the dataset. To overcome this, the nonlinear solution is suggested as alternative to discover the nonlinear behavior in the input space [4]. The idea is the input data matrix is mapped into high dimensional nonlinear feature space through a nonlinear mapping function. Next, these mapped data are used in the post process of data analysis. Several studies with different dataset using the beneficial of applying various types of nonlinear high-dimensional space mapping method have been discussed [see [4, 5, 6, 7, 8, 9]]. This has proven its superiority as the most efficient method than such linear method [8, 10]. Considering the existing nonlinear methods, the kernel is the most used due to its flexibility and efficiency in the computational process [4, 6, 7, 11, 12, 13]. Instantaneously, the kernel solution presented here apply the famous Reproducing Kernel Hilbert Spaces (RKHS) in the mapping formulation to reach the nonlinear optimization procedure [7, 14] with universally result to consistent result [15]. It has been observed so far there is still less number of study considers its robustness particularly with improvement on the resistance diagnostics to the multiple outliers and high leverage points.

The outlier and/or high leverage data points are results of the experimental and/or non experimental error. The outlier is an unwanted observation in the y direction, oppositely the high leverage data point is an outlier in the X direction. These have potential to influence the fitting performance that lead to the misleading result. Using the non-robust statistical methods is not recommended, since the estimated parameter is sensitive to the small changes in the data distribution. Hence, seeking for the robust procedure that may not suffer from the influence is needed.

In 1973, Huber introduced the M-estimator [16] that minimizes the function of residual in the least square estimation. This estimator is well known as robust location parameter estimator which uses $\ell_{1}-norm$ for measuring residual in the observation that is identified as outlier. The M-estimator fails to attain high breakdown point and has unbounded influence factor hence it is not resistant to the leverage points [17, 18]. To improve the low breakdown point, another robust method called S-estimator [17] was proposed by minimizing the scale of residual in M-estimator. In fact, the method still fails to identify the leverage points due to its unbounded influence factor [18]. In compliance to improve the resistance through bounded influence, a Generalized M-estimator (GM-estimator) [19] was suggested. The GM-estimator is still suffered in a loss of efficiency [20] because observations with good leverage point are still down-weighted. Another GM-estimator using Schweppe solution [21] was proposed by adjusting the leverage down-weighted with the size of residual. It was observed, the proposed GM has also less breakdown point (closer to 0), when the number of m predictor increases [22] with some outliers more likely happen. As development, the GM6 was introduced [23] and it has the bounded influence and high breakdown-point (with closely to 50%). The GM6 has benefit in a distinction between outliers and bad high leverage observations. It uses Least Trimmed Square (LTS) [24] as initial estimator than classical Ordinary Least Square (OLS). To fit the limitations of previous robust estimators, the MM-estimator [17] was introduced. The MM-estimator reaches the robustness by discovering the smallest possible dispersion of the residual in S-estimator and continues to find the resistant estimator through the efficiency of the M-estimator [20]. Some robust high breakdown-point estimator with a good efficiency such Least Trimmed Square (LTS) [24] and Least Median Squares (LMS) [25] usually are used as initial estimator in MM-estimator. However, the earlier robust methods effective only to detect the outlier and single high leverage points. In fact, they fail to detect multiple high leverage cases by reason of masking and/or swamping effects on the low leverage some (see [16, 22, 26, 27, 28]). As solution, the Diagnostic Robust Generalized Potential (DRGP) (see [29]) is assigned by initially classify the suspects high leverage points and real outlier using the Robust Mahalanobis Distance (${\text{RM}}_{i}^{2}$) based on Minimum Volume Ellipsoid (MVE) [30, 31]. The DRGP calculates the generalized potentials criteria to each observations whether the suspected observations have potentially high leverage points or not.

The method presented here is the extension work of the previous study (in [7]) that introduces the robust algorithm procedure in the nonlinear Kernel-PLS. The new method is named as a Kernel Partial Diagnostic Robust Potential (KPDRGP) with the parameter estimators are highly resistance to the existing of multiple outlier and high leverage points in the dataset. The study provides a development and important contribution to tackle the challenges of scientific big data especially in vibrational spectroscopy technique. In the paper, some existing robust methods are also reviewed and integrated in the PLS term. Some discussions are provided to compare the methods through simulation using different datasets.

## 2 Some consideration methods

### 2.1 Partial least square regression

Partial Least Squares Regression (PLSR) [2] is the most used statistical method in the Chemometric analysis. Particularly for NIR spectral dataset, the PLSR has an important role to investigate the relationship between two different sets of predictor X and response y. Here, we limited the study only in the case of n>>m, where m represents the number of predictor variable and significantly greater or less than the number of sample size n. Related to the high dimensional problem in the NIR dataset the PLSR becomes a standard procedure to decrease the dimensional of predictor variables X. Technically, the predictor X is projected to a smaller set of uncorrelated variables called new components. The PLSR keeps a maximize covariance of the highly collinear original predictors in these new components and regress these to the single y. PLS has free distribution assumption means it does not matter whatever data distribution is, it is also opposed violations of independence, collinear, and small sample size that are known as main assumptions in regression.

Let consider a multiple regression model consists two different sets of predictor X and single response y, in matrix form these can be written as

(1) y=Xb+e

where y,e are nx1 vector, X is nxm matrix and b is mx1 vector, the common solution of estimator b using OLS method is given by

(2) $$b\;=\;{\left(X^{T}X\right)}^{-1}X^{T}y$$

Earlier it considers the NIR dataset consisting large number of m predictors. So that there will infinite number of solution for estimator b as $X^{T}X$ is singular which not meet the usual trivial theorem on rank. As a special procedure to this case, it is needed to extract new components by maximizing a covariance criterion between predictor X and response y where linking the centered values of these two sets [32].

Using the Eq. (1) and initialize a starting score vector of u from single y, there exists an outer relation for predictor X which can be defined as

(3) $$X\;=\;VP^{T}\;+\;E$$

where P is the matrix mxl consisting loading vector 1xm${\left\{p_{g}\;=\;\left(X^{T}\;v_{g}\right)/\left({v_{g}}^{T}v_{g}\right)\right\}}_{g=1}^{l}$ with $v_{g}$ is the nx1 column vector of scores $x_{j}$ in X ${\left\{v_{g}\;=\;\left(X\;w_{j}\right)/\left(w_{j}^{T}w_{j}\right)\right\}}_{g=1}^{l}$ involve $w_{j}$ as a mx1 vector of weight for X ${\left\{w_{j}\;=\;\left(X^{T}u\right)/\left(u^{T}u\right)\right\}}_{j=1}^{m}$, V is a nxl matrix of the nx1 vector $v_{g}$ and E is a nxm matrix of residual in outer relation for predictor X. Unlike in Principal Component Analysis (PCA), $v_{g}$ is not simply as multiplication of the matrix X and loading matrix P, a correspondence to mxl weight matrix of W is considered. Following the similar procedure as like predictor X, the outer relation for response y also can be given as

(4) $$y\;=\;u\;q^{T}+\;\;f$$

In Eq. (4), variable y is in univariate term, q is the loading lx1 vector${\left\{q_{g}\;=\;\left(\;y^{T}\;v_{g}\right)/\left(v_{g}^{T}v_{g}\right)\right\}}_{g=1}^{l}$, u is nxl matrix of y block score, and f is a nx1 vector of residual in y. u is also called as linear inner relation between X block score and y block score which is calculated as ${\left\{u\;=\;b_{g}v_{g}\;\text{with}\;\;b_{g}\;=\;u^{T}\;v_{g}/\left(v_{g}^{T}\;v_{g}\right)\right\}}_{g=1}^{l}$ or written as

(5) $$u\;=\;V\;b_{inner}\;+\;g$$

where $b_{\text{inner}}$ is a lx1 vector of regression coefficient as solution using LS on the decomposition of vector u, and g is nx1 matrix of residual in the inner relation. Applying the normalization in W, q, P as the process for improving the inner relation and obtaining orthogonal X block scores, so that the mixed relation of PLSR model by integrating Eq. (4) and Eq. (5) can be formed as [2].

(6) $$\begin{array}{c} y\;=\;u\;q^{T}\;+\;\;f\\ y\;=\;V\;b_{inner}\;q^{T}+\;g\;q^{T}\;+\;f\\ y=V\;b_{inner}\;q^{T}\;+\;\overset{⌣}{f}\\ y=V\;a^{T}\;+\;\;\overset{⌣}{f} \end{array}$$

the lx1 vector coefficient a is $a^{T}=b_{inner}\;q^{T}$ and $\overset{⌣}{f}$ denotes nx1 vector of residual in mixed relation $\overset{⌣}{f}=g\;q^{T}+\;f$. In Eq. (6) it holds $a=V^{T}y$, without loss of generality that $X=VP^{T}$ as in Eq. (3), then Eq. (6) can be redefined by multiplying the two sides with mxl weight matrix of W that results

(7) $$\begin{array}{c} y\;=\;X\;W^{*}\;a+\;\;\overset{⌣}{f}\\ {y\;=\;X\;W\;(P^{T}W)}^{-1}\;a+\;\;\overset{⌣}{f} \end{array}$$

with $V\;=\;X\;W^{*}$ and ${W^{*}\;=\;W(P^{T}W)}^{-1}$. Let define $b_{PLSR}\;=\;W\;{\left(P^{T}W\right)}^{-1}a$ as matrix coefficient of mixed relation in PLSR, and then Eq. (7) is equivalent to

(8) $$y\;=\;X\;b_{PLSR}+\overset{⌣}{f}$$

$\overset{⌣}{f}$ need to be minimized. Applying the previous relation Eq. (3) and Eq. (4), it holds $W\;=\;X^{T}U$, $P=X^{T}V{\left(V^{T}V\right)}^{-1}$ such that the estimator for parameter $b_{PLSR}$ can be defined as

(9) $${{\hat{b}}_{PLSR}\;=\;X^{T}u\;(V^{T}X\;\;X^{T}u)}^{-1}\;V^{T}y,\;{\hat{b}}_{PLSR}\in {ℜ}^{mx1}$$

Eq. (5)

$b_{PLSR}$ denotes the l dimensional vector of regression coefficient in PLSR. In the PLSR model, the Non Linear Iterative Partial Least Squares (NIPALS) algorithm is applied to computational.

### 2.2 Partial robust M-Regression

The Partial Robust M-Regression (PRM) is an alternative robust version to classical PLSR [33]. It uses a modified robust M-estimate [16] in PLSR by assigning additional reweighting function $w_{i}$ as diagnostic tool [34]. The PRM succeed to identify the suspected outlier and leverage points both in y and X direction. To maximizing the covariance between the response and predictor variable as basic principle in PLSR, the PRM modifies the covariance function to obtain loadings and scores using a robust procedure.

Assigning proper weighted covariance

$$Cov_{w}\left(v,y\right)\;=\;\frac{1}{n}\sum_{i=1}^{n}\left(w_{i}v_{i}y_{i}\right)$$

Then the loading vectors in X is determined in a sequential way through

$$\begin{array}{l} p_{g}\;=\;\underset{p}{arg\;max}\;\;Cov_{w}\left(X\;p,y\right)\\ \text{s.t}\;\left\|\;p\;\right\|=1 \end{array}$$

$Cov_{w}\left(\;p_{j},p\right)=0$ for all preciously computed $p_{j}$

Continuing the Eq. (8), for 1≤i≤n, the OLS solution of $b_{PLSR}$ for single y is defined as

(10) $${\hat{b}}_{PLSR}\;=\;\underset{b}{arg\;\;min}\;\left(\sum_{i=1}^{n}{\left(y_{i}-v_{i}\;b_{PLSR}\;\right)}^{2}\right)$$

The estimator is optimal if $E\;\left(\overset{⌣}{f}\right)=0$ and $Var\;\left(\overset{⌣}{f}\right)=1$ or where $\overset{⌣}{f}\;∼\;N\;\left(\;0\;,\;1\;\right)$, otherwise fail to satisfy the normal assumption, the OLS losses its optimality, hence the robust estimator yields better.

The robust M-estimates reconstruct the squares in term of u then giving

(11) $${\hat{b}}_{M}\;=\;\underset{b}{arg\;\;min}\left(\sum_{i=1}^{n}\theta \;\left(y_{i}-v_{i}\;b_{M}\right)\right)$$

with $\theta \;\left(u\right)=u^{2}$, $\theta \;\left(y_{i}-v_{i}\;b_{M}\right)={\left(y_{i}-v_{i}\;b_{M}\right)}^{2}$ as θ(u) is defined to be loss function which is symmetric and nondecreasing. Recall the $\overset{⌣}{f}$ as residual nx1 column vector ${\left\{{\overset{⌣}{f}}_{i}=y_{i}-v_{i}\;b_{PLSR}\;\right\}}_{\;i\;=\;1}^{\;n}$ in the objective function of Eq. (11). Applying the partial derivative and reweighing technique [34], then the row weight in each observations is $w_{i}^{r}=\theta \;({\overset{⌣}{f}}_{i})/{{\overset{⌣}{f}}_{i}}^{2}$, taking $\theta \;({\overset{⌣}{f}}_{i})=w{\;}_{i}^{r}\;{{\overset{⌣}{f}}_{i}}^{2}$ thus equation in Eq. (11) can be rewritten as

(12) $${\hat{b}}_{M}\;=\;\underset{b}{arg\;\;min}\;\left(\sum_{i=1}^{n}w_{i}^{r}\;{{\overset{⌣}{f}}_{i}}^{2}\right)$$

The weight in Eq. (12) is considered less sensitive to the vertical outlier without pin-pointing the leverage points in X direction, follows Serneels [33] then another weight $w_{\;i}^{x}$ is added to identify the suspected leverage points. The test criteria is $w_{\;i}^{x}\approx 0$ would be suspected as leverage point. The solution of partial robust M-regression hence can be formulized as

(13) $${\hat{b}}_{PRM}\;=\;\underset{b}{arg\;\;min}\;\left(\sum_{i=1}^{n}w_{i}^{r}\;w_{i}^{x}\;{{\overset{⌣}{f}}_{i}}^{2}\right)$$

where $w_{i}=w_{i}^{r}\;w_{i}^{x}$ is called to be the generalized weight. The Eq. (13) can be rewritten as

(14) $${\hat{b}}_{PRM}\;=\;\underset{b}{arg\;\;min}\;\left(\sum_{i=1}^{n}w_{i}\;{{\overset{⌣}{f}}_{i}}^{2}\right)$$

here, the weight $w{\;}_{i}^{r}$ and $w{\;}_{i}^{x}$ are calculated as

(15) $$w{\;}_{i}^{r}\;=\;\rho \;\left(\frac{{\overset{⌣}{f}}_{i}}{\hat{\sigma }},c\right)$$

with $\hat{\sigma }$ uses robust $MAD\;\left({\overset{⌣}{f}}_{1},\ldots ,{\overset{⌣}{f}}_{n}\right)=\underset{i}{median}|{\overset{⌣}{f}}_{i}-\underset{j}{median\;\;{\overset{⌣}{f}}_{j}|}$, ρ(z,c) is a weight function [34] and constant c follows the Huber's function [16].

(16) $$w{\;}_{i}^{x}=\rho \;\left(\frac{\left\|\;\;v_{i}-med_{L1}\left(V\right)\;\right\|}{median\;\;\left\|\;v_{i}-med_{L1}\left(V\right)\;\right\|},c\right)$$

‖⋅‖ is Euclidean norm, $med_{L1}\left(V\right)$ is a robust estimator of the center of the l dimensional score vectors, and $v_{i}={\left(v_{i,1},\ldots ,v_{i,l}\right)}^{\;T}$ is the vector of component score matrix V that need to be estimated.

## 3 Proposed method

### 3.1 Partial robust MM-Regression

Extending the previous work in PRM [33], the improved robust MM-regression [17] was proposed to determine the final estimates in the PLSR. The estimator combines a high breakdown point (50%) of a class of LTS-estimators and better efficiency of modified M-estimators [33]. Using LTS-estimators [31] on PLSR as

(17) $${\hat{b}}_{LTS}\;=\;\underset{b}{arg\;\;min}\;\;{\hat{\sigma }}_{LTS}\;\left({\overset{⌣}{f}}_{1},\;{\overset{⌣}{f}}_{2},\ldots ,\;{\overset{⌣}{f}}_{n}\right)$$

the solution in Eq. (17) is obtained as it finds the minimal possible dispersion of residual. Recall the procedures in the classical MM-estimators: 1) the M-estimators are used as initial estimates; 2) calculate the scale estimates ${\hat{\sigma }}_{LTS}$ (LTS-estimators) [31] using the obtained residual ${\overset{⌣}{f}}_{i}$ with objective function labeled as $\theta_{0}$; 3) proceed the M-estimators with objective function $\theta_{1}$ to calculate the final estimates. The improved on robust partial MM-regression is now the M-estimators replaced with the modified M-estimators. The aims are beside of having high breakdown point and more efficient estimator, the final estimator is resistant to the outliers and high leverage points. Following the Eq. (14), by applying the re-descending score function $\psi_{1}\left(u\right)=\frac{\partial \;\theta_{1}\left(u\right)}{\partial \;u}$ with $\left\{u\;=\;\frac{y_{i}-v_{i}\;{\hat{b}}_{M}}{{\hat{\sigma }}_{LTS}}\right\}$ and value of the scale estimates ${\hat{\sigma }}_{LTS}$ the solution for partial robust modified MM-estimators is the slight modification on weight $w{\;}_{i}^{r}$ which is

(18) $$w{\;}_{i}^{r}\;=\;\rho \;\;\left(\frac{{\overset{⌣}{f}}_{i}}{{\hat{\sigma }}_{LTS}},c\right)$$

with the additional resistant weight $w_{\;i}^{x}$ as in Eq. (16). The objective function $\theta_{1}$ does not need to be similar to the $\theta_{0}$, but should satisfy the properties as below:

i. θ is symmetric and continuously differentiable, with θ(0)=0
ii. There exists a>0 such that θ is rigidly increasing on [0  ,  a] and constant on [a  ,  ∞)
iii. $\theta_{1}\left(\overset{⌣}{f}\right)\leq \theta_{0}\left(\overset{⌣}{f}\right)$ hence,

$$\sum_{i=1}^{n}\theta_{1}\;\left(\frac{y_{i}-v_{i}\;{\hat{b}}_{M}}{{\hat{\sigma }}_{LTS}}\;\right)\leq \sum_{i=1}^{n}\theta_{0}\;\left(\frac{y_{i}-v_{i}\;{\hat{b}}_{M}}{{\hat{\sigma }}_{LTS}}\right)$$

### 3.2 Partial robust GM6-Regression

The GM-estimator was introduced by Mallows [19] and Schweppe [21] with objective to improve the resistance of classical M-estimator to the suspected high leverage point through downweighing procedure. The general robust GM-estimator using equation in Eq. (1) is given as

(19) $${\hat{b}}_{GM}\;=\;\underset{b}{arg\;\;min}\;\left(\sum_{i=1}^{n}w_{i}\;\theta \;\left(\frac{y_{i}-v_{i}\;\hat{b}}{z_{i}\;\hat{\sigma }}\right)\;\right)$$

where θ is a specified function which is absolutely continuous and non-decreasing on [0  ,  ∞),$w_{i}$, $z_{i}$ represent the weights and depend on the matrix of V that are updated iteratively from initial estimate using any robust method, and $\hat{\sigma }$ is an estimate of robust scale. Taking the $\psi \left(\overset{⌣}{f}\right)=\frac{\partial \;\theta \left(\overset{⌣}{f}\right)}{\partial \;\overset{⌣}{f}}$, the GM-estimator in Eq. (19) then yields the solution as

(20) ∑i=1nwiψ(yi−vibˆzi σˆ)vi=0

the Mallows solution [19] provides only the weight $w_{i}$ (ranged from 0 to 1), that is another weight $z_{i}=1$. The weight $w_{i}$ is a square root function of the diagonal elements of the hat matrix$\left\{\;\;w_{i}\propto {\left(1-h_{ii}\right)}^{\;1/2}\;\right\}$; $H\;=V{\left(V^{T}V\right)}^{\;-1}V^{T}$ [21]. It reduces the involvement of observations with high influential points in the factor space. However, the estimator results in loss efficiency because the good leverage points are also downweighed. As improvement, the Schweppe [21] suggested the solution by adjusting the leverage weights function based on the size of residual ${\overset{⌣}{f}}_{i}$ (in PLSR) with now the weight $z_{i}=w_{i}$; $\left\{w_{i}\propto \frac{\sqrt{1-h_{ii}}}{r_{i}}\psi \left(\frac{r_{i}}{\sqrt{1-h_{ii}}}\right)\right\}$; $\left\{r_{i}=\frac{{\overset{⌣}{f}}_{i}}{\hat{\sigma }}\right\}$ [35] and $\hat{\sigma }$ as scale estimator in [36]. Although the strategy is better than Mallows, the Schweppe solution is problematic with low breakdown point [17] and it is not consistent when the residuals are asymmetric [37]. Moreover, it is suffered with the influence of multiple outliers in the factor space [36]. To overcome these limitation, the GM6 [23] with high breakdown point (closer to 50%) and bounded influence was introduced. The advantage of GM6 is it can classify whether the suspected leverages are good or bad. In the algorithm, the initial estimates $b_{0}$ uses high breakdown (BDP = 50%) LTS estimator [31] with the scale estimate is calculated using LMS estimator [31] which provides 0.95 efficiency$\left\{\;\hat{\sigma }=1.4826\;\;\left(1+5/\left(n-p\right)\right)\;Median\;\;\left|r_{\;i}\right|\;\right\}$. The partial GM6 estimator can be defined as

(21) $${\hat{b}}_{GM6}\;=\;{\hat{b}}_{0}\;+\;{\left[\;\sum_{i=1}^{n}v_{i}^{T}Β\;v_{i}\right]}^{-1}\;\times \;\sum_{i=1}^{n}\hat{\sigma }\;W\;\psi \left(\frac{r_{i}\;({\hat{b}}_{0})}{\hat{\sigma }\;w_{i}}\right)\;v_{i}$$

it is known that the hat matrix $h_{ii}$ fails to prevent the masking effects of multiple outliers in the factor space [38], the weighing strategy $W=diag\;\;\left(w_{i}\right)$ using Robust Mahalanobis Distance (${\text{RM}}_{i}^{2}$) [30] then is preferred

(22) $$w_{i}\;\propto min\;\left(1,\frac{\chi_{0.95,p}^{2}}{{\text{RM}}_{i}^{2}}\right)$$

(23) $${\text{RM}}_{i}^{2}\;=\;{\left(v_{i}-m_{v}\right)}^{\;T}C_{v}^{-1}\left(v_{i}-m_{v}\right)$$

with $m_{v}$ and $C_{v}$ are robust location and shape estimates of the minimum volume ellipsoid (MVE) estimators. The value of MVE is calculated from the matrix of latent variables V, $B\;=\;diag\;\;\left(\psi '\left(\frac{r_{i}({\hat{b}}_{0})}{\hat{\sigma }\;w_{i}}\right)\right)$ as diagonal in the derivative of Huber's function ψ, and $\chi_{0.95,p}^{2}$ is the suitable chi-square distribution. In the GM6, the final estimates are calculated in the single step (Newton Raphson) rather than iteratively. Here, as alternative to S-estimator [36] the MM-estimator with better asymptotic efficiency is assigned as initial estimator.

### 3.3 Partial robust DRGP-Regression

As a cause of masking and/or swamping effect in the factor space, almost the existing robust methods only resistant to the influence of single outlier or high leverage point. With multiple high influential points in the factor space they are ineffective and fail to identify [22, 30, 39]. This may result to a poor fitting process, multicollinearity, and heteroscedastic [40]. Due to this problematic, Habshah [29] introduced the DRGP procedure in order to determine whether the observations potentially have multiple high leverage point or not. The procedure uses suitable robust cut-off point proposed by Imon [41] on the calculated generalized potentials $p_{ii}^{*}$ to decide the suspected observations. Here, the robust DRGP is applied in the PLSR by assigning the new component variables V as input space instead of original data X.

Recall the multiple regression model in Eq. (1), the easiest way to detect the influence point of outlier is based on their residual e. Using the OLS method, the term of the true disturbance vector function of leverage and true error weight matrix W can be defined as

(24) $$e\;=\;y-\hat{y}=\left(1-V\;{\left(V^{T}V\right)}^{-1}V^{T}\right)\;e$$

where $W=V\;{\left(V^{T}V\right)}^{-1}V^{T}$ reflects joint effect of m predictors on the fitted values $\hat{y}$ and it is also known as hat matrix. The diagonal elements $w_{ii}$ is the hat values in matrix W and it is considered as a measure of leverage values of y corresponds to $\hat{y}$.

The DRGP consists of two steps; the first step is to determine the suspected high leverage points using robust approach in Eq. (23), with robust cut-off criteria [39].

(25) $$Median\;\;\left({\text{RM}}_{i}^{2}\right)+3\;MAD\;\left({\text{RM}}_{i}^{2}\right)$$

this involves the calculation of median and Median Absolute Deviation (MAD) in the ${\text{RM}}_{i}^{2}$. The second step uses the diagnostic approach called generalized potential denoted as $p_{ii}^{*}$ for all members in data set to confirm the suspicion. Let R as a set of “remaining” good observations and D as a set of “deleted” observations, then the R consists of (n−d) observations after d<(n−k) observations in D are deleted. In a case where the ith observations are deleted from the remaining set of R and further joins the deletion set in D. For any such i, the $w_{ii}^{-\left(D+i\right)}$ equals to $w_{ii}^{-\left(D\right)}/\left(1-w_{ii}^{-\left(D\right)}\right)$ as shown below

(26) $$w_{ii}^{-\left(D+i\right)}=v_{i}^{T}{\left(V_{R}^{T}V_{R}\right)}^{-1}x_{i}+\frac{{\left(v_{i}^{T}{\left(V_{R}^{T}V_{R}\right)}^{-1}v_{i}\right)}^{2}}{1-v_{i}^{T}{\left(V_{R}^{T}V_{R}\right)}^{-1}v_{i}}=\frac{w_{ii}^{-\left(D\right)}}{\left(1-w_{ii}^{-\left(D\right)}\right)}$$

for a case when the size of R is (n−1) and D=i the $w_{ii}^{-\left(D\right)}$ is equivalent as an natural extension of ith potentials $p_{ii}$ in ${\text{RM}}_{i}^{2}$ [30] as $w_{ii}^{\left(-i\right)}=v_{i}^{T}{\left(V_{\left(i\right)}^{T}V_{\left(i\right)}\right)}^{-1}v_{i}=p_{ii}$, thus $w_{ii}^{-\left(D\right)}$ is the ith diagonal element in matrix $V\;\;{\left(V_{R}^{T}V_{R}\right)}^{-1}V^{T}$.

The generalized potentials criteria as in [41] is given as

(27) $$p_{ii}^{*}\;=\;\{\begin{array}{c} \frac{w_{ii}^{-\left(D\right)}}{1-w_{ii}^{-\left(D\right)}}fori\in R\\ w_{ii}^{-\left(D\right)}\;fori\in D \end{array}$$

with the robust cut-off point consider both the dimension of the predictors and any account of the number of observations,

(28) $$p_{ii}^{*}>Median\;\;\left(p_{ii}^{*}\right)+c\;MAD\;\;\left(p_{ii}^{*}\right)$$

here c is a constant value of 2 or 3 and $MAD\;\;\left(p_{ii}^{*}\right)=Median\;\;\left\{\left|p_{ii}^{*}-Median\;\;\left(p_{ii}^{*}\right)\right|\right\}/0.6475$. The criteria test, if the $p_{ii}^{*}$ of suspicion is greater than the cut-off point in Eq. (28) the suspicion in the first step is true. While if it is not, then put back the observation into the dataset and recalculate the generalized potentials $p_{ii}^{*}$ on the remaining subset.

### 3.4 Kernel - PLS

With the restriction of nonlinear relationship and improper spectra signature in the NIR dataset, the use of kernel function as solution is suggested. By relating the RKHS and feature space F in [7], each point in the original input vectors are mapped nonlinearly to a higher dimensional F. Recall the NIPALS algorithm in PLSR model, the kernel method replaces the dot products calculation of elements in the mapped data corresponding to a nonlinear function in the original input space. Consequently, the orthogonal weight vectors w and a as in PLSR cannot be estimated directly, hence the NIPALS algorithm should be reconstructed into its nonlinear kernel-based variant [42].

Some general definitions associating with the theory in RHKS by Aronszajn (see in [43]) are provided as below:

Definition 1 (*Inner product*)*. Let* H *be a vector space over field* ℜ*. A function* ${\left\langle \cdot ,\cdot \right\rangle }_{H}\text{:}H\times H\to ℜ$ *is said to be an inner product on* H *must satisfy the conditions*

i. Bilinearity: ${\left\langle \alpha \;f+\beta \;g,k\right\rangle }_{H}=\alpha {\left\langle \;f,k\right\rangle }_{H}+\beta {\left\langle \;g,k\right\rangle }_{H}\forall \;f,g,k\in H,\;\forall \;\alpha ,\beta \in ℜ$
ii. Symmetric: ${\left\langle f,g\right\rangle }_{H}={\left\langle \;g,f\right\rangle }_{H}\forall \;f,g\in H$
iii. Positive definiteness: ${\left\langle f,f\right\rangle }_{H}\geq 0,\forall \;f\in H$ and ${\left\langle f,f\right\rangle }_{H}=0$ if and only if f=0.

Definition 2 (*Normed space*) *is a linear* (*vector*) *space* H *over field* ℜ*on which a norm on* H *is defined and for* f,g∈H*and* ∀ α∈ℜ *satisfies the properties*

i. Positive homogeneity: ‖α f‖=|α|  ‖f‖  
ii. Triangle inequality: ‖f+g‖≤‖f‖+‖g‖
iii. Positive definiteness: ‖f‖≥0 and ‖f‖=0 if and only if f=0

Definition 3 (*Convergent*)*. Let* H *be an inner product space, a sequence* ${\left\{f_{n}\right\}}_{n=1}^{\infty }$ *is said to be Convergent if for* ∀ε  >0 *there exists an element* f∈H*, such that* $\left\|\;f-f_{n}\right\|\;\;<\varepsilon$ *as*n→∞*.*

Definition 4 (*Cauchy sequence*)*. Let* H *be an inner product space, a sequence* ${\left\{f_{n}\right\}}_{n=1}^{\infty }$ *be a Cauchy sequence in* H *if* ∀ε  >0 *there exists a positive integer* N*, such that* $\left\|\;f_{n}-f_{m}\;\right\|\;<\varepsilon$ *whenever* n,m≥N*.*

Any convergent sequence is also a Cauchy sequence.

Definition 5 (*Complete*)*. A linear* (*vector*) *space* H *is said to be complete if the Cauchy sequence is convergent.*

Definition 6 (*Hilbert Space*)*. A normed space is said to be a Hilbert space on which the norm is induced by an complete inner product space* ⟨f,g⟩ *as the relation* ${\left\|f\right\|}_{H}\text{:}\;=\sqrt{\;{\left\langle f,f\right\rangle }_{H}}$*.*

Definition 7 (*Kernel*)*. Consider* X *be an arbitrary non-empty set. A function* K:X×X→ℜ *is said to be a kernel if* ℜ*-Hilbert space and a map* ϕ:X→H *are exist, such that* $\forall \;\;x_{i},x_{i}^{T}\in X$*,*

(29) $$K\;\left(\;x_{i},{x_{i}}^{T}\right)\text{:}={\left\langle \phi \left(x_{i}\right),\phi {\left(x_{i}\right)}^{T}\right\rangle }_{H}$$

Definition 8 (Reproducing Kernel Hilbert Space). Let H be a Hilbert space of function on non-empty set X whose ⟨f,g⟩ as complete inner product space and${\left\|f\right\|}_{H}\text{:}\;=\sqrt{\;{\left\langle f,f\right\rangle }_{H}}$ as the normed space in H, for f,g∈H. Reproducing kernel of H is said to be a function of K:X×X→ℜ if satisfies the properties

i. $K_{x}\left(\cdot \right)=K\left(x,\;\cdot \right)\in H$ for any x∈X, where
ii. Reproducing property: $f\left(x\right)={\left\langle f\left(\cdot \right),K\left(x,\;\cdot \right)\;\right\rangle }_{H}$, for any f∈H and x∈X.

Applying the function $K_{x}$ at y to the properties (ii) in (Def.8) giving

$$f\left(x\right)={\left\langle f\left(y\right),K\left(x,y\right)\right\rangle }_{H},\;\text{for}\;\forall \;f\in H,\;\text{and}$$

$$K_{x}\left(y\right)=\left\langle K_{x},K_{y}\right\rangle ,\;\text{for}\;x,y\in X$$

with the symmetric function of two variables in K satisfying the Mercer's theorem [44].

Theorem 1 *Let* H *be a Hilbert space of function over non-empty set* X*. The following properties are equivalent*

1. H has a reproducing kernel
2. For any x∈X, the function $\phi_{x}\text{:}H\to ℜ$ defined by $\phi_{x}\left(f\right)=f\left(x\right)$ is continuous.

Following Mercer's theorem, each positive definite kernel K(x,y) on a compact domain X×X may be written as

(30) $$K\left(x,y\right)\;=\;\sum_{i=1}^{s}\lambda_{i}\phi_{i}\left(x\right)\;\phi_{i}\left(y\right),\;S\leq \infty$$

where $\lambda_{i}$ is the sequence of eigenvalues with $\lambda_{1}\geq \lambda_{2}\geq \ldots \geq \lambda_{S}$ of K(x,y) and ${\left\{\phi_{i}\right\}}_{i=1}^{s}$ is the infinite sequence of eigenfunctions. Moreover, Eq. (30) can be reformulated as

(31) $$K\left(x,y\right)\;=\;\sum_{i=1}^{s}\sqrt{\lambda_{i}}\phi_{i}\left(x\right)\;\sqrt{\lambda_{i}}\;\phi_{i}\left(y\right)=\left(\phi \left(x\right)\cdot \phi \left(y\right)\right)=\left(\;\phi {\left(x\right)}^{T}\cdot \phi \left(y\right)\;\right)$$

It is obvious to see that any kernel K(x,y) in Eq. (31) also corresponds to a canonical (Euclidean) dot product in a possibly higher dimensional feature space F, with the mapping function

(32) $$\begin{array}{l} \phi \text{:}X\to F\\ x\to \left(\sqrt{\lambda_{1}}\phi_{1}\left(x\right),\sqrt{\lambda_{2}}\phi_{2}\left(x\right),\ldots ,\sqrt{\lambda_{s}}\phi_{s}\left(x\right)\right) \end{array}$$

with $\left\{\;{\left\{\sqrt{\lambda_{i}}\phi_{i}\left(x\right)\right\}}_{\;i=1}^{\;\;s},x\in X\right\}$ denotes as feature mapping.

Based on these properties, the description of the nonlinear kernel is discussed. Assuming a nonlinear transformation function of the input variables $x_{i}\in X\subset {ℜ}^{m}$ into the feature space F.

(33) $$\phi \text{:}x_{i}\in X\subset {ℜ}^{m}\to \phi \left(x_{i}\right)\in F$$

with ϕ(⋅) is a nonlinear mapping function in the input space of the original input vectors to the feature space F. The mapping ϕ replaces $x_{i}$ with the $\phi \left(x_{i}\right)$ and result to the high-dimension which can even be infinite feature space F. Once the component score matrix V in linear PLSR is obtained, then a nonlinear PLSR is computed as the original input data space. Define ϕ as an nxs matrix of mapped space data with the ith row is the vector $\phi \left(x_{i}\right)$ in s dimensional feature space F. Here the use of nonlinear kernel function is preferred instead of an explicit nonlinear mapping. The Eq. (31) giving the deflation

(34) $$K=\phi \;\phi^{T}$$

with K represents as nxn kernel Gram matrix of the cross dot products among all mapped input data points ${\left\{\;\phi \left(x_{i}\right)\right\}}_{i=1}^{n}$. Using the normalized component scores $v_{g}$, the deflation of K and y are formulized as

(35) $$\begin{array}{l} \phi_{g+1}\;\phi_{g+1}^{T}\leftarrow \left(\phi_{g}-v_{g}v_{g}^{T}\phi_{g}\right)\;{\left(\phi_{g}-v_{g}v_{g}^{T}\phi_{g}\right)}^{\;T}\\ K_{g+1}\leftarrow \left(I-v_{g}v_{g}^{T}\right)\;\;K_{g}\;\left(I-v_{g}v_{g}^{T}\right) \end{array}$$

and

(36) $$y_{g+1}\leftarrow y_{g}-v_{g}v_{g}^{T}y_{g}=y_{g}\left(I-v_{g}v_{g}^{T}\right)$$

the coefficient matrix of the KPLS regression model in F then can be written as

(37) $${\hat{b}}_{KPLS}\;=\;\phi^{T}u\;{\left(V^{T}K^{T}U\right)}^{-1}\;V^{T}y,\;b_{KPLS}\in {ℜ}^{mxp}$$

and the final prediction of component concentration in the regression model is given as

(38) $$\begin{array}{l} \hat{y}\;=\;\phi \;\phi^{T}u\;{\left(V^{T}\phi \phi^{T}\;u\right)}^{-1}\;V^{T}y\\ {\hat{y}}_{v}\;=\;K_{v}u\;{\left(V^{T}K\;u\right)}^{-1}\;V^{T}y \end{array}$$

where $\hat{y}$ and ${\hat{y}}_{v}$ are the prediction of calibration set and validation set, respectively. $K_{v}$ is the $n_{v}x\;n\;$ kernel matrix of validation set with elements are composed of $K_{ij}=K\left(x_{i},x_{j}\right)$, which are input vectors of calibration set ${\left\{\;x_{i}\right\}}_{i=n+1}^{n+n_{v}}$ and validation set ${\left\{\;x_{j}\right\}}_{j=1}^{n}$. As suggested by Wu [45, 46], centering the kernel both in calibration and validation are very important to provide the bias term to be zero. Centralization on the mapped data in F simply can be calculated as

(39) $$\begin{array}{l} K\;=\left(I-\frac{1}{n}1_{n}1_{n}^{T}\right)\;K\;\left(I-\frac{1}{n}1_{n}1_{n}^{T}\right)\\ K_{v}\;=\left(K_{v}-\frac{1}{n}1_{n_{v}}1_{n}^{T}K_{v}\right)\left(I-\frac{1}{n}1_{n}1_{n}^{T}\right) \end{array}$$

with I represents as n-dimensional identity matrix, and $1_{n},1_{n_{v}}$ denotes the vectors whose elements equal to 1, with lengths n and $n_{\text{v}}$, respectively.

### 3.5 Kernel - PRM

The Kernel-PRM (simply called as KPRM) develops the concept of PRM to incorporate the nonlinear feature in the original input factor dataset. In the prior study [47], the KPRM uses kernel function to transform the nonlinear relationship in the original input space into a linear PLS through high-dimensional feature space. The method provides generalized weight $w_{i}$ scheme as robust procedure to the nonlinear kernel to remove the outlier and downgrade the influence of bad high leverage point in the dataset. A modified PRM as proposed in the KPRM is the input matrix X is subsequently replaced as the outer product $\phi \;\phi^{\text{T}}$ of the nxn kernel Gram matrix K. A main concern in the KPRM procedure is based on the weighing scheme in K. Let define $\overset{⌣}{K}$ as the weighted matrix of K, so that

(40) $$\overset{⌣}{K}=\left(\Omega \phi \right)\;{\left(\Omega \phi \right)}^{T}=\Omega K\Omega$$

where Ω is said to be the diagonal weight matrix, with the ith elements in the diagonal matrix equals to the generalized weight in the PRM.

### 3.6 Kernel Partial Diagnostic Robust Potential

Another alternative of robust method in the class of nonlinear kernel called Kernel Partial Diagnostic Robust Potential (KPDRGP) is proposed. The KPDRGP follows the principles of the Partial Robust DRGP-Regression that uses the suitable robust cut-off point on the calculated generalized potentials $p_{ii}^{*}$ to confirm the suspected high leverage. Instead of applying the explicit nonlinear mapping of X to the feature space F, the kernel function $\phi \;\phi^{T}$ as the cross dot products among all mapped input data points ${\left\{\;\phi \left(x_{i}\right)\right\}}_{i=1}^{n}$ is used. Here, a Gaussian kernel function is applied due to its superiority to handle nonlinear dataset [48].

(41) $$K_{i,j}=K\left(\;x_{i},x_{j}\right)=exp\left(-\frac{\left\|\;x_{i}-x_{j}\;\right\|{\;}^{2}}{\sigma^{2}}\right)$$

where $\sigma^{2}$ is the non-robust variance of original training data X and ‖ ⋅ ‖ is Euclidean norm of two matrices.

Let define $\overset{⌣}{\text{R}}{\text{M}}_{i}^{2}$ as robust Mahalanobis distance that employ the ith elements kernel Gram matrix K in the input space, such that

(42) $$\;\overset{⌣}{\text{R}}{\text{M}}_{i}^{2}={\left({\overset{⌣}{v}}_{i}-{\overset{⌣}{m}}_{v}\right)}^{\;T}C_{v}^{-1}\left({\overset{⌣}{v}}_{i}-{\overset{⌣}{m}}_{v}\right)$$

where ${\overset{⌣}{m}}_{v}$ and ${\overset{⌣}{C}}_{v}$ are robust location and shape estimates of the minimum volume ellipsoid (MVE) estimators. These robust estimator are calculated from the matrix of new components $\overset{⌣}{V}$ with kernel Gram matrix K in the input space. To justify the suspicion high leverage points it is important to determine how $w_{ii}^{-\left(D\right)}$ is adjusted. Define new $w_{ii}^{-\left(D\right)}$ as the ith diagonal element in matrix $\overset{⌣}{v}\;\;{\left({\overset{⌣}{v}}_{R}^{T}{\overset{⌣}{v}}_{R}\right)}^{-1}{\overset{⌣}{v}}^{T}$ and result to the new generalized potential ${\overset{⌣}{p}}_{ii}^{*}$. This value then is used as diagnostic tool to confirm the suspicion through cut-off point in Eq. (28).

(43) $${\overset{⌣}{p}}_{ii}^{*}=\{\begin{array}{c} \frac{{\overset{⌣}{w}}_{ii}^{-\left(D\right)}}{1-{\overset{⌣}{w}}_{ii}^{-\left(D\right)}}fori\in R\\ {\overset{⌣}{w}}_{ii}^{-\left(D\right)}\;fori\in D \end{array}$$

### 3.7 Algorithm Kernel Partial Diagnostic Robust Potential

Let define $K=\phi \left(X\right)\;\phi {\left(X\right)}^{T}$ be the kernel Gram matrix of the cross dot products among all mapped input data points ${\left\{\;\phi \left(\;x_{i}\right)\right\}}_{i=1}^{n}$ in feature space F. In addition, K must be centered, and y need to be normalized to satisfy the normal assumption.

1. Apply a nonlinear mapping function $\left\{\;\phi \text{:}x_{i}\in X\subset {ℜ}^{m}\to \phi \left(x_{i}\right)\in F\;\right\}$ in the input space of the original input vectors to the feature space F. The mapping ϕ replaces $x_{i}$ with the $\phi \left(x_{i}\right)$ and result to the high-dimensional feature space F.
2. Replace the outer product $\phi \left(x_{i}\right)\;\phi {\left(x_{i}\right)}^{T}$ by the kernel matrix K. Apply an identity matrix I as the initial weight Ω on the kernel matrix to get the weighted ${\overset{⌣}{K}}_{g}$ and output vector ${\overset{⌣}{y}}_{g}$, ${\overset{⌣}{y}}_{g}=\Omega \;\;y_{g}$.
3. Initialize the weighted output vector $\overset{⌣}{y}$ as a column weighted latent variable ${\overset{⌣}{u}}_{1}={\overset{⌣}{y}}_{g}$.
4. Regress ${\overset{⌣}{K}}_{g}$ on ${\overset{⌣}{u}}_{1}$ to obtain the weight ${\overset{⌣}{w}}_{g}=\left(\;{\overset{⌣}{K}}_{g}^{T}\;{\overset{⌣}{u}}_{1}\right)/\left(\;{\overset{⌣}{u}}_{1}^{T}\;{\overset{⌣}{u}}_{1}\right)$, then normalize ${\overset{⌣}{w}}_{g}$ and rename it as ${\overset{⌣}{v}}_{g}={\overset{⌣}{w}}_{g}/\left\|{\overset{⌣}{w}}_{g}\right\|$. ${\overset{⌣}{v}}_{g}$ is the weighted of new component variables with kernel Gram matrix ${\overset{⌣}{K}}_{g}$ in the input space.
5. Calculate the weights in ${\overset{⌣}{y}}_{g}$ by ${\overset{⌣}{c}}_{g}=\left(\;{\overset{⌣}{y}}_{g}^{T}{\overset{⌣}{v}}_{g}\right)/\;{\overset{⌣}{v}}_{g}^{T}{\overset{⌣}{v}}_{g}$ and project the ${\overset{⌣}{y}}_{g}$ on the ${\overset{⌣}{c}}_{g}$ to calculate the new component variable in ${\overset{⌣}{y}}_{g}$ using ${\overset{⌣}{u}}_{g}^{*}={\overset{⌣}{y}}_{g}\;\;{\overset{⌣}{c}}_{g}$ and normalize ${\overset{⌣}{u}}_{g}^{*}$ .
6. Determine the ${\overset{⌣}{y}}_{g}$ and ${\overset{⌣}{v}}_{g}$ improvement using ${\overset{⌣}{u}}_{\text{ Δ}}={\overset{⌣}{u}}_{g}^{*}-{\overset{⌣}{u}}_{g}$, if ${\overset{⌣}{u}}_{\text{ Δ}}>\;\;\text{tol}$, go to step 1 to 5 until convergence using ${\overset{⌣}{u}}_{g}^{*}$. While if ${\overset{⌣}{u}}_{\text{ Δ}}<\;\;\text{tol}$, the first latent PLS is found and proceed to the step 7. The tol refer to tolerance limit for convergence which is set to 10^−4^.
7. Deflate the ${\overset{⌣}{K}}_{g}$ and ${\overset{⌣}{y}}_{g}$ using ${\overset{⌣}{K}}_{g+1}=\left(\;1-{\overset{⌣}{v}}_{g}{\overset{⌣}{v}}_{g}^{T}\right)\;\;{\overset{⌣}{K}}_{g}\left(\;1-{\overset{⌣}{v}}_{g}{\overset{⌣}{v}}_{g}^{T}\right)$, ${\overset{⌣}{y}}_{g+1}={\overset{⌣}{y}}_{g}\left(\;1-{\overset{⌣}{v}}_{g}{\overset{⌣}{v}}_{g}^{T}\right)\;$
8. Continue to step 4 until all l PLS components are determined.
9. Here, the new calculated $\overset{⌣}{V}$ as nonlinear PLSR are used as the new input space in the robustness procedures.
10. For each i point on $\left({\overset{⌣}{v}}_{i},{\overset{⌣}{m}}_{i}\right)$ pair, calculate the $\overset{⌣}{\text{R}}{\text{M}}_{i}^{2}$
11. Use the cut-off point criteria $Median\;\left(\overset{⌣}{\text{R}}{\text{M}}_{i}^{2}\right)+3MAD\;\left(\overset{⌣}{\text{R}}{\text{M}}_{i}^{2}\right)$ to determine the suspected outlier and high leverage points in each i point of $\overset{⌣}{\text{R}}{\text{M}}_{i}^{2}$.
12. Calculate the generalized potential values for each observation. If the ${\overset{⌣}{p}}_{ii}^{*}$ of suspicion is less than the cut-off point then put back the observation into the dataset and then recalculate the generalized potentials ${\overset{⌣}{p}}_{ii}^{*}$ on the remaining subset R.
13. The observations that are identified as real outlier and high leverage points are removed from the remaining subset R.
14. Update the weight Ω by assigning the weight score as 0 to the deleted observation, while the rest observations in the subset R are weighted as 1.
15. Continue to step 2 to recalculate the new weighted ${\overset{⌣}{K}}_{g}$ and output vector ${\overset{⌣}{y}}_{g}$, re-perform the rest steps until all PLS estimates are obtained.

## 4 Results and discussion

**Figure 1:**
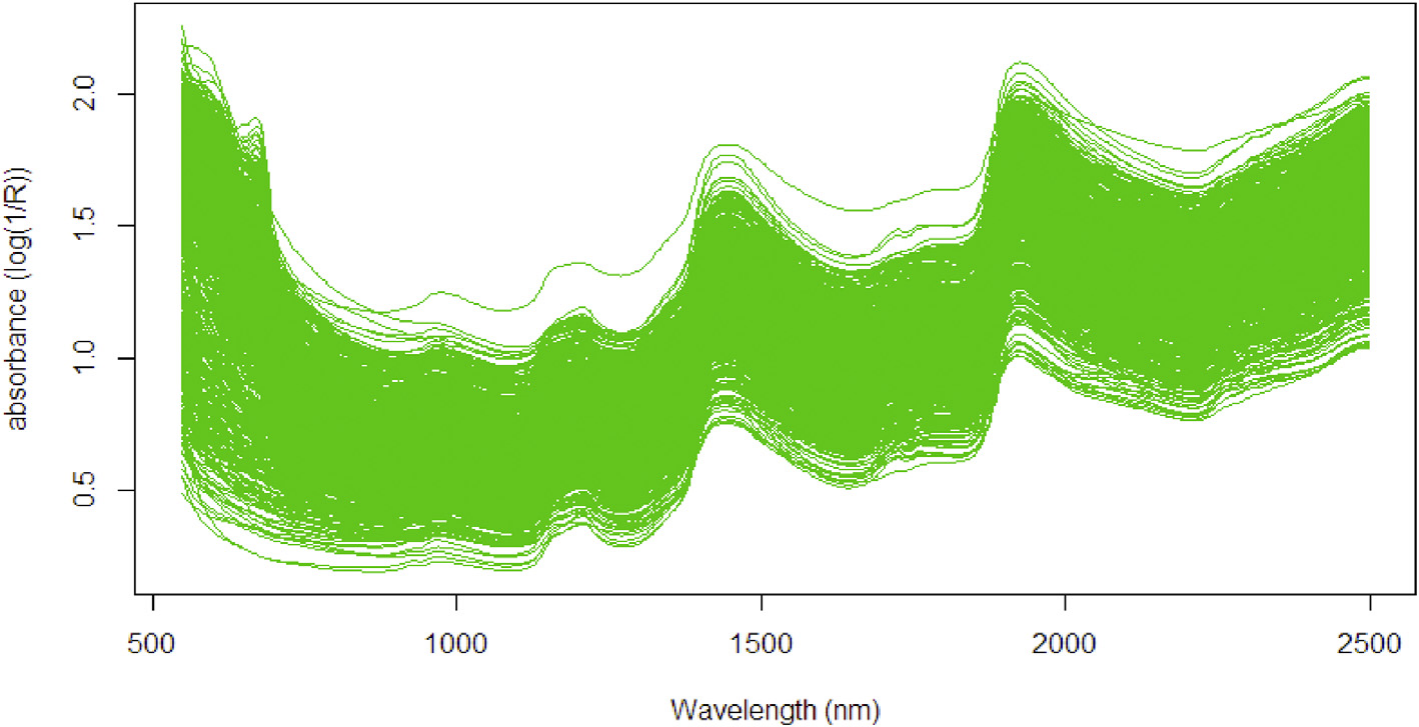
First run of averaged spectra on oil palm fresh fruit mesocarp.

To examine the superiority, all the proposed methods: PLS, PRM, PRMM, PRGM6, PRDRGP, KPLS, KPRM, and KPDRGP were evaluated in order using the simulation data and real data. The simulation data uses the sin (x) function with added noise e∼N(0,0.05), while the real data uses NIR spectral dataset of oil palm (*Elaeis guineensis*) fresh fruit mesocarp (see Figure 1). This fruit samples are a part of breeding trial experiment in Palapa Estate, PT. Ivomas Tunggal, Riau Province, Indonesia. There are two response variables observed from the fruit mesocarp sample: percent of oil to dry mesocarp (%ODM) and percent of oil to wet mesocarp (%OWM). The spectral data were collected (in contact) using the QualitySpec Trek from Analytical Spectral Devices (ASD Inc., Boulder, Co., USA) on 12 different sampling positions in the fruit bunch (without destructive). These data are good enough to evaluate the non linear effect of the proposed methods. The comprehensive algorithms about the proposed methods were written into R-language using Ri386 software (http://r-project.org) with 3.4.2 version for windows.

### 4.1 Simulation data

The simulation data uses the sin (x) function to generate the nonlinear behavior dataset. Earlier it was known that some existing multivariate calibration only capable to capture the linear datasets, otherwise it fails to capture the dataset structure with highly nonlinear. In this section, 41 samples data were randomly generated in the range of [0,10] as training set and were calculated with correspond to the sine function in Eq. (44). To evaluate the sensitivity of the proposed method, 7 random outliers were manually added in the training set. The outliers were marked orderly followed their sample number which are 10, 12, 18, 19, 28, 33, and 36.

(44) f(x)=sin(−3/4x)

**Figure 2:**
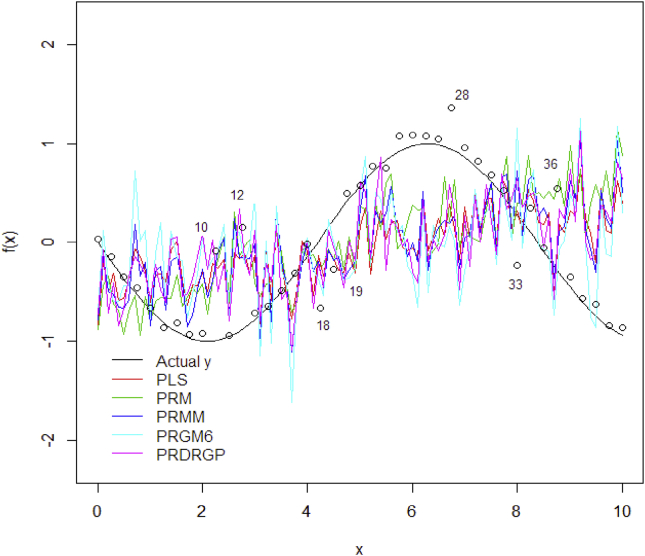
Predictions on testing data using training model of non-kernel methods.

**Figure 3:**
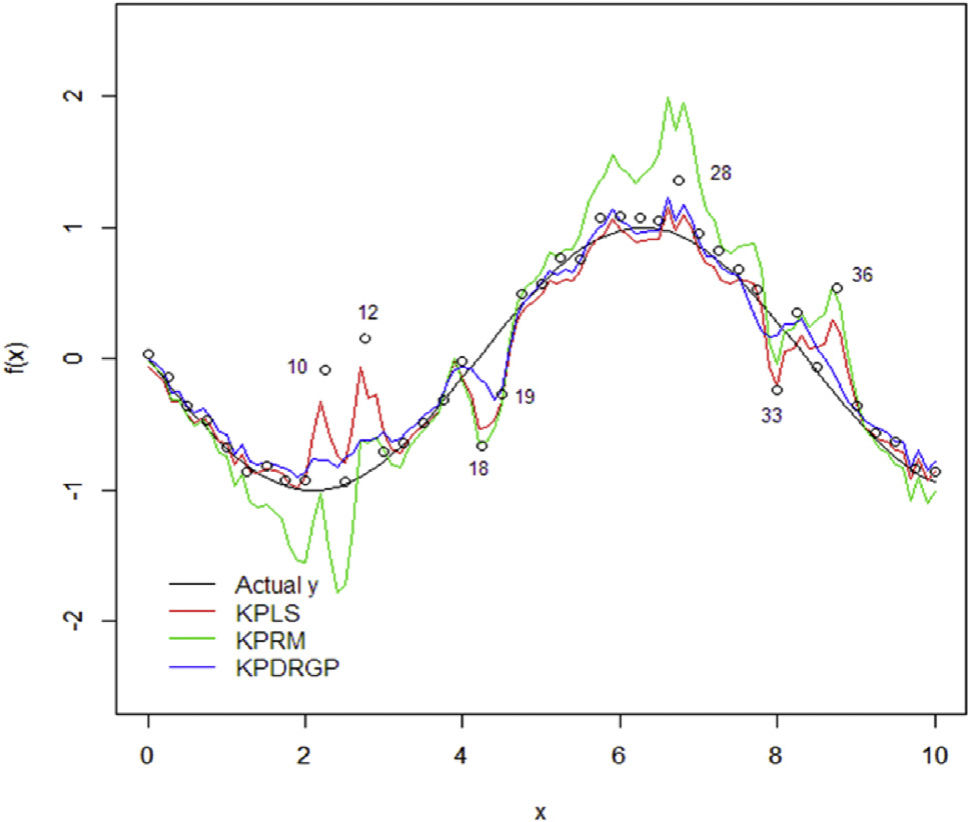
Predictions on testing data using training model of kernel methods.

To test the consistency in the training model, 101 samples was also uniformly generated using the range of [0,10] as testing set. The performance of the methods both using non-kernel and kernel methods are presented in Figure 2 and Figure 3.

**Figure 4:**
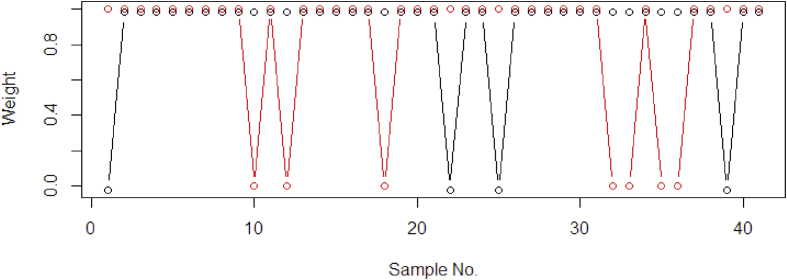
The final sample weight values using DRGP algorithm: PRDRGP (black) and KPDRGP (red).

As seen in Figure 3, by applying the kernel transformation on the methods the prediction results is really promising. The methods succeed to capture the structure of highly nonlinear dataset. While without kernel transformation (see Figure 2), all the methods fails to fit the model. The proposed KPDRGP method shows a better fitting line compared with the KPLS and KPRM. With the contamination of outliers in the training dataset, the KPDRGP is less sensitive and succeeds to decrease the influence. It is interested to evaluate the robustness of the proposed DRGP procedure with and without kernel transformation in PLSR through their final weight values (see Figure 4).

With the influence of nonlinear effect in the dataset, the linear PRDRGP algorithm fails to identify the true outliers in the dataset (see Figure 4). While by transforming the data into a high dimensional feature space using kernel Hilbert Space method, it succeeds to capture the true outliers in the dataset. The KPDRGP method decides the sample number: 10, 12, 18, 32, 33, 34, and 36 were the true outliers. However it is still failed to screen the remaining 2 outliers (sample number: 19 and 34), this might be caused by the extraction process in the data transformation. In Figure 3, the KPDRGP also succeed to prevent the over-lower prediction in the testing data. The summarization of the prediction results both using training and testing dataset can be seen in Table 1. Here, the statistical measures uses Desirability Indices [49] such as Root Mean Squared Error (RMSE), Coefficient of Determination (${\text{R}}^{2}$), Ratio of Prediction to Deviation (RPD), Bias, and Standard Error (SE).

**Table 1:** Statistical measures on prediction results using sine function simulation data.

Dataset	Desirability Index	PLS	PRM	PRMM	PRGM6	PRDRGP	KPLS	KPRM	KPDRGP
Training	RMSE	0.631	0.690	0.645	0.662	0.654	0.302	0.265	0.101
	${\text{R}}^{2}$	0.201	0.109	0.171	0.141	0.156	0.920	0.958	0.980
	RPD	1.117	1.021	1.092	1.064	1.077	1.087	2.656	5.724
	Bias	-0.106	0.003	-0.062	-0.081	-0.084	0.002	0.026	0.001
	SE	0.639	0.698	0.653	0.671	0.662	0.320	0.269	0.150
Testing	RMSE	0.647	0.704	0.686	0.768	0.713	0.235	0.679	0.143
	${\text{R}}^{2}$	0.133	0.107	0.104	0.029	0.104	0.854	0.891	0.965
	RPD	1.065	0.979	1.004	0.897	0.966	2.928	1.015	4.819
	Bias	0.032	0.089	0.049	0.028	0.049	0.024	0.114	0.054
	SE	0.650	0.707	0.690	0.772	0.717	0.236	0.682	0.144

In Table 1, by preventing the effects of outlier in the fitting process, the KPDRGP produces the lowest prediction error (SE and RMSE) both in training and testing data set are about 0.150 and 0.144, respectively. The method shows the highest ${\text{R}}^{2}$ both in training and testing dataset which are 0.980 and 0.965, respectively. This ${\text{R}}^{2}$ value represents the variability for a response variable that can be explained by predictor variables. The method also shows better reliability value (RPD) with less bias than the other methods. In the class of kernel, the KPLS method is inferior due to contamination from outliers in the dataset. By downgrading the effect of multiple outliers in the dataset, the KPDRGP is superior to KPRM.

### 4.2 Oil to dry mesocarp data

**Figure 5:**
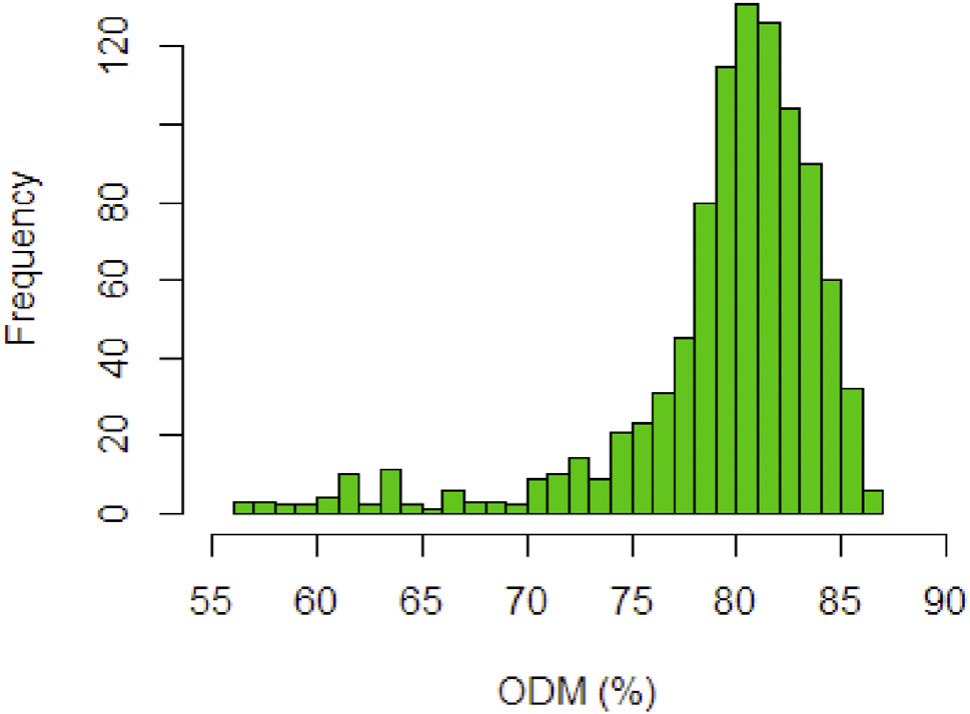
Frequency distribution histogram of %ODM values.

The real NIR spectra dataset on fresh fruit mesocarp was used to examine the robustness of the kernel solution. The dataset contains 960 observations and 489 wavelengths data points as predictor variables. In this section, the %ODM was used as response variable. As seen in Figure 1, the higher spectral absorbance corresponds to a higher %ODM in fruit mesocarp. Figure 5 shows the frequency distribution of the %ODM values used as training set. To build a good training model, the training set should contain all possible values. Earlier, it was considered if the dataset has high dimensional problem as result of huge number of observation and predictor variables. This condition may risk to the multicollinearity that lead to the heteroscedastic problem in the residual. To encounter this, the PLSR method is preferred to summarize the covariance between predictor variable and the response variable by downscaling the original variables X into less number of new PLS components V.

In this study, the maximum of PLS components used in the model were limited to 20 and 30 components. This strategy was assigned to evaluate the efficiency of PLSR model using less and large number of PLS components. The assumption is the less number of PLS components used in the training model the most efficient is. As seen in Table 2 it is observed that in the class of non-kernel method, all the proposed methods (PLS, PRM, PRMM, PRGM6, PRDRGP) are not better than the class of kernel partial method (KPLS, KPRM, KPDRGP). The KPDRGP method produces the lowest prediction error (SE and RMSE) even using less and higher number of PLS which are about 0.466 and 0.204, respectively. The reliability of the training model based on its RPD is also greater than 3; means that the training model constructed arestrong enough to prove the reliability for routine analysis. With contamination of outliers in the dataset, the KPLS fails to prevent the influence hence result to a higher SE with lower ${\text{R}}^{2}$ compared to the KPRM and KPDRGP.

**Table 2:** Statistical measures on prediction results using %ODM dataset.

PLS	Desirability Index	PLS	PRM	PRMM	PRGM6	PRDRGP	KPLS	KPRM	KPDRGP
l = 20	RMSE	3.305	3.344	3.474	3.601	3.343	0.617	0.561	0.466
	${\text{R}}^{2}$	0.556	0.578	0.562	0.548	0.570	0.925	0.988	0.993
	RPD	1.551	1.533	1.475	1.423	1.519	8.299	9.073	11.588
	Bias	0.240	0.298	0.426	0.498	0.313	0.011	-0.021	-0.004
	SE	3.307	3.346	3.476	3.603	3.325	0.618	0.561	0.466
l = 30	RMSE	2.934	2.992	3.166	2.652	2.969	0.273	0.242	0.203
	${\text{R}}^{2}$	0.632	0.660	0.631	0.633	0.665	0.937	0.997	0.998
	RPD	1.747	1.713	1.619	1.932	1.726	18.804	19.911	25.579
	Bias	0.060	0.133	0.385	0.196	0.142	0.014	0.018	0.001
	SE	2.996	2.993	3.168	2.954	2.971	0.273	0.242	0.204

**Figure 6:**
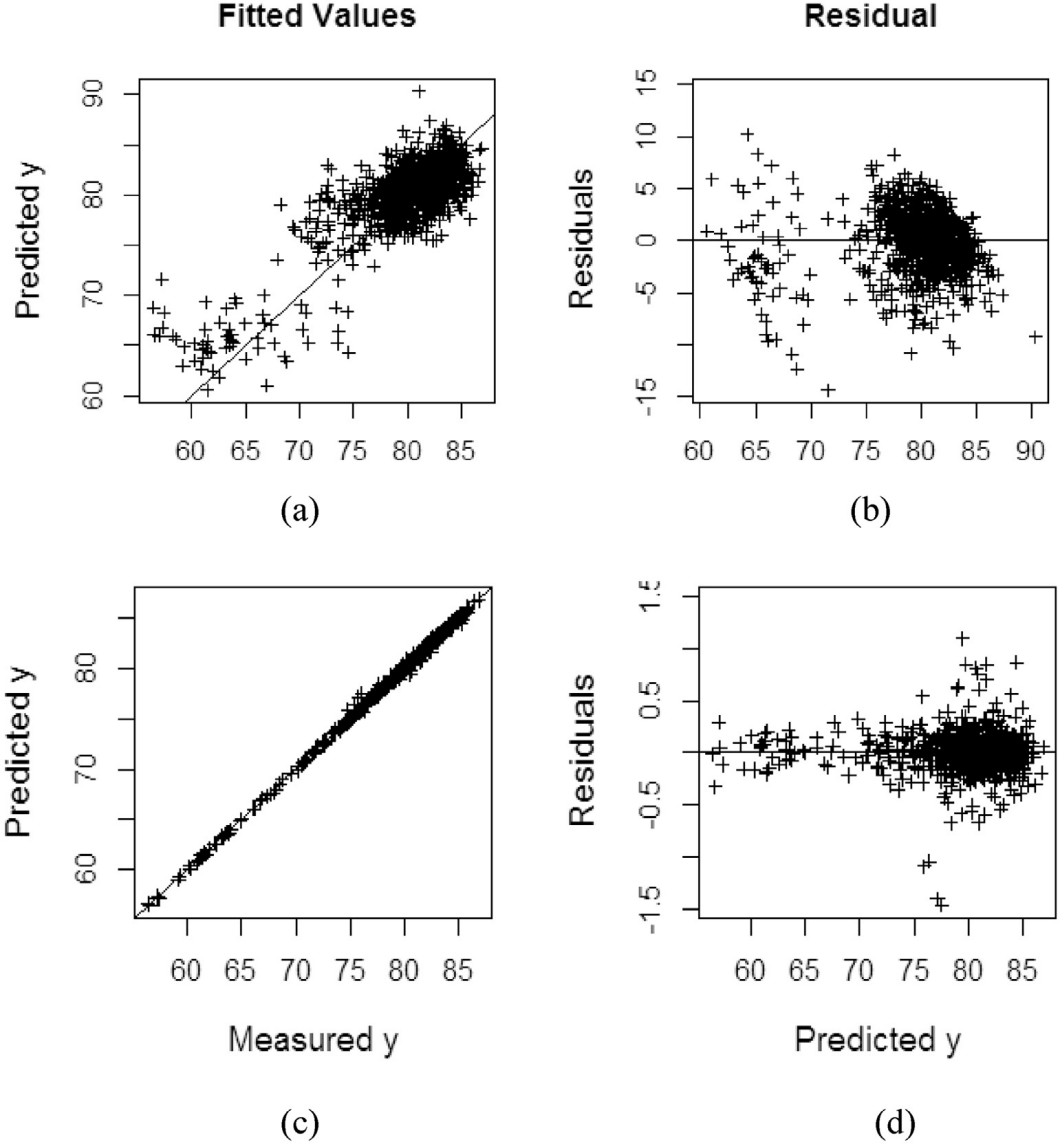
Fitting and residual performance on %ODM dataset: (a) measured vs predicted values on PRDRGP; (b) predicted vs residual on PRDRGP; (c) measured vs predicted values on KPDRGP; (d) predicted vs residual on KPDRGP.

The fitting performance between measured and predicted values using PRDRGP and KPDRGP methods on %ODM dataset are shown in Figure 6. It highlights how well the kernel solution on the proposed KPDRGP (Figure 6(c) and 6(d)) can improve the prediction result using training model (see Table 2). This can be inspected through how close the predicted values are to the fitted regression line using without kernel (Figure 6(a)) and with kernel (6(c)) solution applied in the PRDRGP model. In the study, the PRDRGP fits the data adequately (with ${\text{R}}^{2}$> 95%) compared to the others class of robust PLS such as PRM, PRMM, and PRGM6. When the kernel solution is applied, the accuracy in the KPDRGP increases. The KPDRGP (Figure (6(d)) yields the lowest prediction residual which is closed to 0 than PRDRGP (Figure 6(b)).

### 4.3 Oil to wet mesocarp data

**Figure 7:**
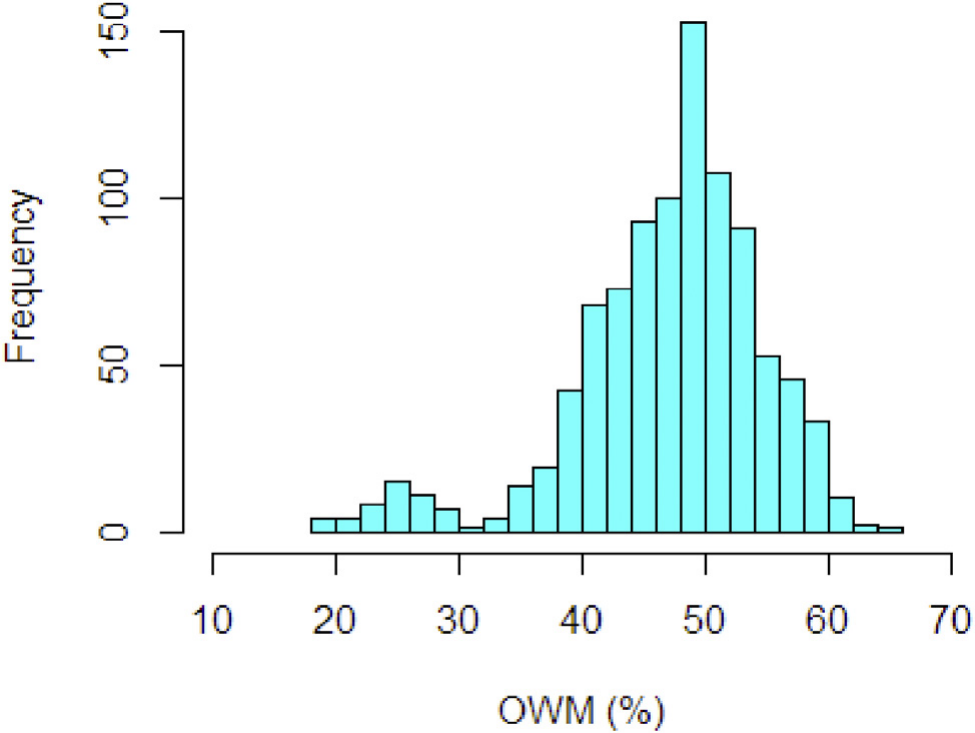
Frequency distribution histogram of %OWM values.

Using similar NIR spectral dataset of oil palm fresh fruit mesocarp as predictor variable, in this section the %OWM (see Figure 7) was assigned as response variable. As seen in Figure 7, the distribution of the response variable covers all possible value of oil content in the wet mesocarp. The maximum number of PLS components was set to 20 and 30 variables. In Table 3, the result shows that the class of non-kernel partial methods which are PRDRGP, PRM, and PRGM6 generally superior to PLS and PRMM. The methods yield a higher ${\text{R}}^{2}$ with lower prediction error (SE and RMSE). According to their reliability index using RPD value, all the non-kernel methods fail to meet (RPD <3). The proposed KPDRGP method still provides the highest fitted regression line (${\text{R}}^{2}$>95%) with lowest prediction error even using less and higher predictors. The proposed robust KPDRGP is also more reliable (with RPD>3) than the other methods. With the possible contamination of outliers in the dataset, the non-robust KPLS fails to prevent the effect hence results to low accuracy compared to the KPRM and KPDRGP.

**Table 3:** Statistical measures on prediction results using %OWM dataset.

PLS	Desirability Index	PLS	PRM	PRMM	PRGM6	PRDRGP	KPLS	KPRM	KPDRGP
l = 20	RMSE	4.471	4.516	4.535	4.487	4.517	0.923	0.768	0.686
	${\text{R}}^{2}$	0.644	0.658	0.655	0.662	0.658	0.956	0.990	0.993
	RPD	1.726	1.709	1.702	1.720	1.709	8.363	9.972	11.646
	Bias	-0.029	0.151	0.004	0.002	0.007	-0.026	0.031	0.003
	SE	4.473	4.519	4.537	4.489	4.519	0.923	0.768	0.686
l = 30	RMSE	4.085	4.126	4.249	4.180	4.130	0.404	0.374	0.279
	${\text{R}}^{2}$	0.710	0.714	0.697	0.712	0.714	0.967	0.998	0.999
	RPD	1.890	1.870	1.816	2.097	1.869	19.098	20.946	28.252
	Bias	0.075	0.048	0.011	-0.030	0.097	0.003	-0.010	0.002
	SE	4.087	4.129	4.251	4.082	4.133	0.404	0.374	0.279

**Figure 8:**
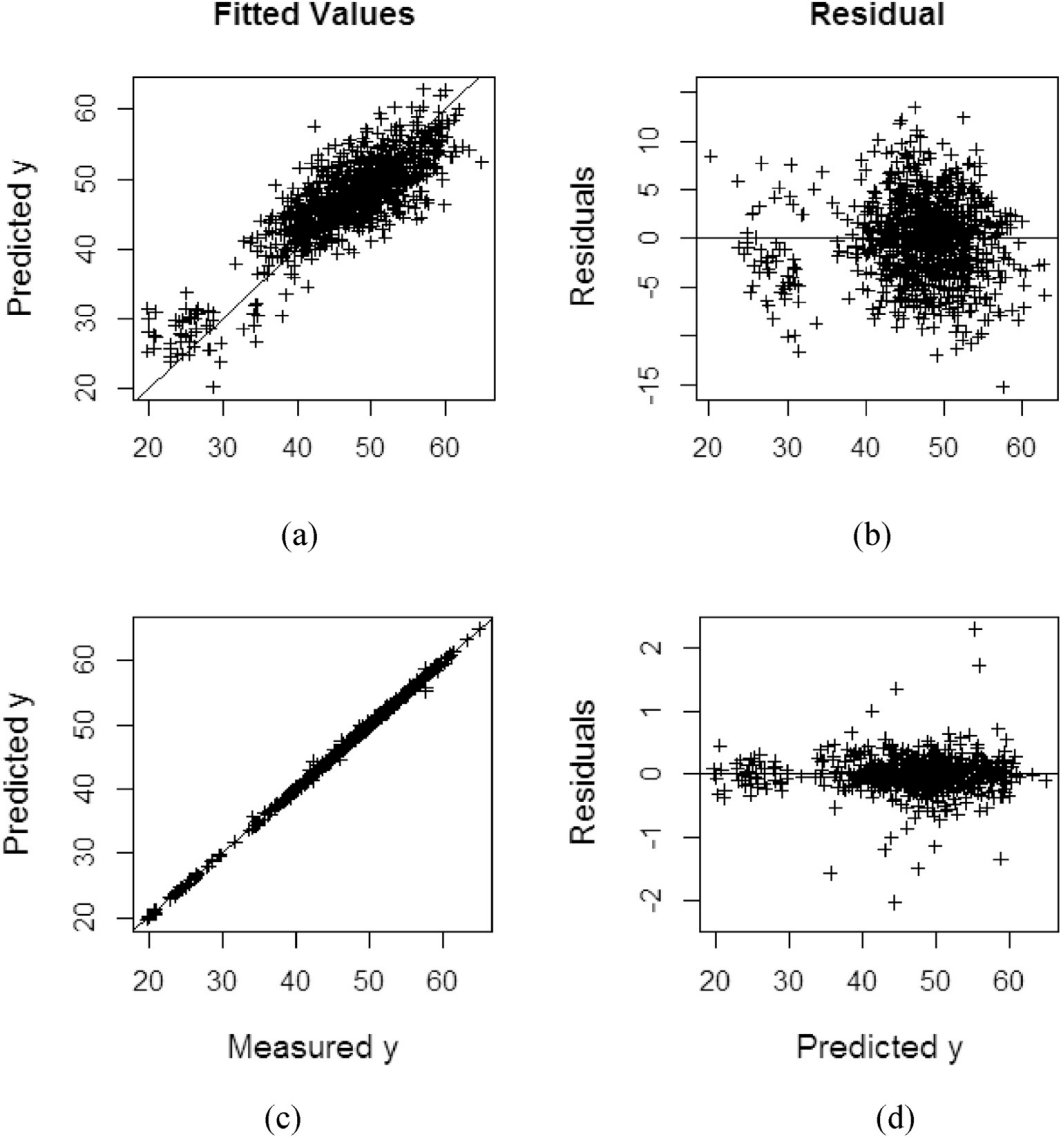
Fitting and residual performance on %OWM dataset: (a) measured vs predicted values on PRDRGP; (b) predicted vs residual on PRDRGP; (c) measured vs predicted values on KPDRGP; (d) predicted vs residual on KPDRGP.

Without kernel transformation applied in the input variable, all the class of non-kernel methods fail to fit the training model. This also lead to produce high bias in the predicted residual (see Table 2) compared to their kernel version. The proposed robust PRDRGP method also does not fit the data adequately (see Figure 8(a) and 8(b)) and still produce high bias (see Table 2). While, by integrating kernel transformation in the model, the method (KPDGP) improved the accuracy and reduce the bias (see Figure 8(c) and 8(d)). This is to confirm that using different dataset, in the class of robust with and without kernel method, the proposed robust PLSR using DRGP procedure is still superior.

## 5 Conclusions

The development of the robust DRGP algorithm in the PLSR method with kernel solution in the input space to handle the high dimensional problem and irregular data space has been discussed. Based on the study using simulation and real NIR spectral dataset, the proposed KPDRGP method was superior compared to the other upgraded methods using some considered robust methods. The KPDRGP succeed to capture the highly nonlinear relationship between the predictor variables against response variables through high-dimensional feature mapping. The reliability of the method also was evaluated; the result also shows the high promising using the method for routine prediction. With the contamination of the multiple outliers and high leverage points in the dataset, KPDRGP was not suffered otherwise the method prevents the influence during the fitting process.

## Declarations

### Author contribution statement

DIVO DHARMA SILALAHI: Conceived and designed the experiments; Performed the experiments; Analyzed and interpreted the data; Wrote the paper.

Habshah Midi: Conceived and designed the experiments; Performed the experiments; Analyzed and interpreted the data; Contributed reagents, materials, analysis tools or data; Wrote the paper.

Jayanthi Arasan, Mohd Shafie Mustafa: Conceived and designed the experiments; Analyzed and interpreted the data.

Jean-Pierre Caliman: Conceived and designed the experiments; Contributed reagents, materials, analysis tools or data.

### Funding statement

This work was supported by the Southeast Asian Regional Center for Graduate Study and Research in Agriculture (SEARCA), Philippines.

### Competing interest statement

The authors declare no conflict of interest.

### Additional information

No additional information is available for this paper.
